# Type I Interferons in the Pathogenesis of Tuberculosis: Molecular Drivers and Immunological Consequences

**DOI:** 10.3389/fimmu.2017.01633

**Published:** 2017-11-27

**Authors:** Meg L. Donovan, Thomas E. Schultz, Taylor J. Duke, Antje Blumenthal

**Affiliations:** ^1^The University of Queensland Diamantina Institute, The University of Queensland, Translational Research Institute, Brisbane, QLD, Australia

**Keywords:** *Mycobacterium tuberculosis*, type I interferon, innate immune signaling, pattern recognition receptors, immune responses, cytokines, patients, mouse models

## Abstract

Tuberculosis (TB) remains a major global health threat. Urgent needs in the fight against TB include improved and innovative treatment options for drug-sensitive and -resistant TB as well as reliable biological indicators that discriminate active from latent disease and enable monitoring of treatment success or failure. Prominent interferon (IFN) inducible gene signatures in TB patients and animal models of *Mycobacterium tuberculosis* infection have drawn significant attention to the roles of type I IFNs in the host response to mycobacterial infections. Here, we review recent developments in the understanding of the innate immune pathways that drive type I IFN responses in mycobacteria-infected host cells and the functional consequences for the host defense against *M. tuberculosis*, with a view that such insights might be exploited for the development of targeted host-directed immunotherapies and development of reliable biomarkers.

## Introduction

With an estimated 1.8 million TB-related annual deaths worldwide, approximately one-third of the global population harboring latent *Mycobacterium tuberculosis* infection, and significant numbers of drug-resistant cases, there is an urgent and compelling need for more sophisticated diagnostic and treatment options for tuberculosis (TB) (1). *M. tuberculosis* is an intracellular pathogen that mainly resides within macrophages. The multi-tiered immune response elicited upon *M. tuberculosis* infection is complex, and our understanding of the requirements for protective immunity remain incomplete (2). Observations in humans and mouse models have firmly established essential roles for interleukin-12 (IL-12) and interferon gamma (IFN-γ)-mediated T cell functions in the control of *M. tuberculosis* infection. Inflammatory cytokines such as tumor necrosis factor alpha (TNF-α) and IL-1 are critical contributors to the immune defense against *M. tuberculosis* (2). Recently described gene signatures in blood of patients with active TB disease (3–9) are being explored extensively for their utility as biomarkers for the reliable diagnosis of active TB, tracking of at-risk individuals, and monitoring of treatment outcome. Additionally, the discovery of IFN-related gene signatures in patients with active TB disease (3–9) has created significant momentum behind investigation of the innate immune pathways and pathophysiological consequences of type I IFN expression during *M. tuberculosis* infection. While IFN-γ is the sole type II IFN, type I IFNs in humans comprise several IFN-α subtypes, IFN-β, IFN-ω, IFN-ε, IFN-τ, and IFN-κ (10). All known type I IFNs signal through a common receptor, IFNAR, which consists of the low-affinity IFNAR1 and the high-affinity IFNAR2 (11, 12). It is increasingly appreciated that type I IFNs not only play a significant role in the antiviral response but are also a central aspect of the host response to bacterial infections (13, 14). In this context, type I IFNs appear to promote or impair pathogen control and disease pathology depending on the infectious agent, acute or chronic state of the infection, and possibly also the model system studied (15). Evidence from human case reports and animal models suggests complex contributions of type I IFNs to the host response during *M. tuberculosis* infection as both protective and detrimental roles for the host have been described. The impact of type I IFNs and related immune response networks on pathology and pathogen control during *M. tuberculosis* infection are also assessed with an eye to host-directed interventions in TB. In this review, we highlight recent advances in the study of TB patient blood gene expression signatures, and the definition of innate immune drivers and immunological consequences of type I IFN expression in the context of *M. tuberculosis* infection.

## Blood Gene Expression Signatures in the Quest for TB Biomarkers

There are increasing numbers of cross-sectional and longitudinal studies exploring whole blood gene expression in TB patients as a potential diagnostic indicator of disease status, manifestation, and responsiveness to treatment [reviewed in more detail elsewhere, e.g. (16–18)]. While the individual genes identified across the various studies are remarkably discordant (18), overall signatures returned from many of these studies have implicated IFN signaling (5, 16). However, independent meta-analyses of publicly available datasets have expressed different views on the dominance and robustness of IFN signatures in active TB (6, 19). An overarching goal of current studies is the identification of minimal gene signatures in global gene expression profiles that can be utilized as reliable TB diagnostic markers in clinical settings. Potential applications of this approach include distinguishing active TB from latent *M. tuberculosis* infection and other infections; monitoring of treatment success/failure; and predicting risk of developing active disease (6–9, 20–22). Some, but not all, of these proposed minimal marker combinations contain IFN-regulated genes. The “omics” approaches currently being pursued clearly indicate innate and adaptive immune response signatures that significantly enhance our current understanding of peripheral host responses during active and latent TB. Consideration of whether or not these molecular signatures are unique to TB is important when designing and interpreting global host response profiles for the derivation of reliable diagnostic markers. Consideration of potential confounding factors, as well as inclusion of unrelated disease controls cohorts, is also critical for success in these endeavors.

Human immunodeficiency virus (HIV) remains a major risk factor for development of active TB. Approximately 1/3 of HIV-positive individuals worldwide are latently infected with *M. tuberculosis* (1). Currently, 55% of notified TB cases are HIV-positive and approximately 22% of TB-related deaths occur in HIV-positive individuals (1). Due to significant virus-associated immunological alterations in HIV-positive individuals, the utility of minimal gene signatures in distinguishing active from latent TB may be compromised in these cases (9, 21). Encouragingly, signatures that distinguish active TB from latent TB in HIV-positive and -negative individuals are emerging (8, 23). Additionally, type II diabetes is being increasingly recognized as a significant comorbidity that adversely affects TB severity, treatment responsiveness, and outcome in high TB burden countries (24). Thus, exploration of molecular signatures to better understand the nature of the TB/type II diabetes interaction is an important upcoming challenge. Notably, a recent study that compared blood transcriptomics in South-Indian TB patients with and without type II diabetes concluded that the utility of blood gene expression markers in the diagnosis of TB might not be confounded by type II diabetes (25). Independent confirmation of these findings in populations where high type II diabetes and high TB burden intersect will be of great significance for the development of new molecular marker-based TB diagnostics.

Interferon-associated genes are present not only in blood gene expression signatures reported for active TB, but also in patients with the granulomatous diseases meliodosis and sarcoidosis (7, 26–29). This suggests that peripheral blood gene expression signatures may be shaped by responses to tissue pathology rather than a specific underlying cause. Moreover, Blankley et al. highlight that severity of clinical symptoms in TB patients is associated with the magnitude of gene expression, regardless of the site of infection (7). Nevertheless, comparisons of global response patterns in active and latent TB with other infectious or inflammatory conditions have yielded encouraging results, as they suggest that molecular signatures that reliably discriminate active TB can be identified (3, 21, 28, 30). Patient cohort-based profiling in search of reliable TB diagnostics calls for careful study design, inclusion of clinical metadata, and clear definition of patient and control cohorts (17). Continuing developments in the analysis of “omics” data, including statistical frameworks, machine learning, multi-platform integration, and network analyses, will be important assets in defining molecular markers that perform robustly independent of age, gender, ethnicity, and comorbidities.

## Molecular Pathways That Drive Type I IFN Production in Mycobacteria-Infected Host Cells

**Figure 1:**
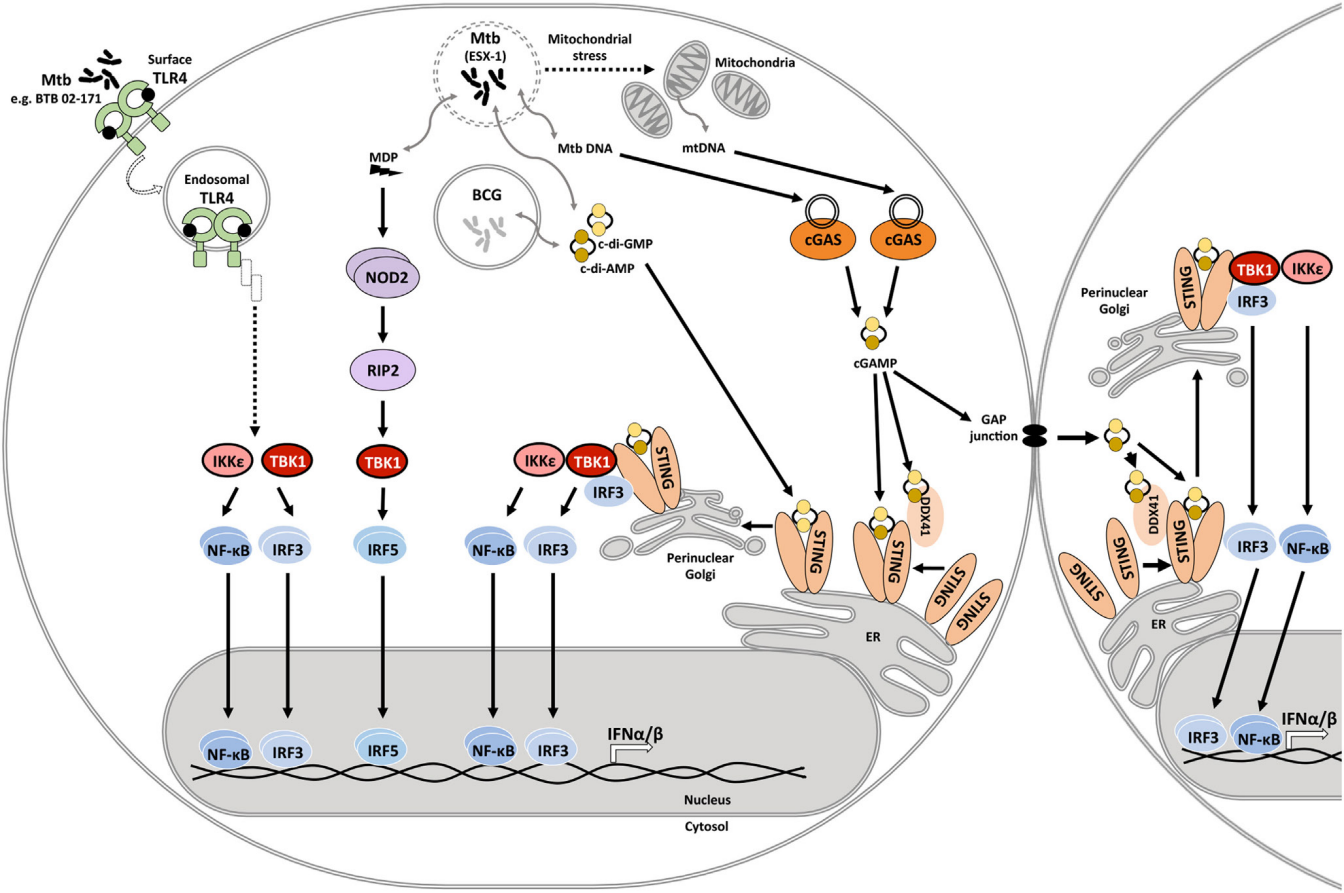
Innate immune signaling pathways that drive type I IFN responses in mycobacteria-infected host cells. During infection, the *Mycobacterium tuberculosis* ESX-1 secretion system facilitates disruption of the phagosomal membrane contributing to mitochondrial stress and leakage of mycobacterial products into the cytosol. These products include the cyclic dinucleotides cyclic diadenosine monophosphate (c-di-AMP) and cyclic diguanosine monophosphate (c-di-GMP), DNA, and *N*-glycolylated muramyl dipeptide (MDP). Recognition of cytosolic mitochondrial and mycobacterial DNA by cyclic GMP-AMP synthase (cGAS) initiates formation of the second messenger cyclic-GMP-AMP (cGAMP). cGAMP and bacterial cyclic dinucleotides interact with dimeric STING on the endoplasmic reticulum or the STING-accessory molecule DDX41. STING activation and relocation to the perinuclear Golgi initiates recruitment and activation of TANK-binding kinase 1 (TBK1), and possibly also IKKε. Subsequent activation and nuclear translocation of the functionally active dimeric transcription factors interferon regulatory factor (IRF)3 and NF-κB drives expression of type I IFNs. cGAMP may also access uninfected cells *via* gap junctions, triggering STING activation and type I IFN expression in bystander cells. MDP is recognized by NOD2, which leads to RIP2-mediated activation of TBK1, culminating in IRF5 dimerization and nuclear translocation. *M. tuberculosis* isolate BTB 02-171 induces type I IFN expression dependent on TLR4. It is inferred that the induction pathway is similar to the known toll-like receptor (TLR)4 endosomal signaling pathway. However, this awaits experimental confirmation. *Mycobacterium bovis* Bacillus Calmette–Guérin (BCG) lacks ESX-1, yet, can elicit type I IFN expression. Cyclic dinucleotide-mediated STING activation independent of ESX-1 has been described.

*Mycobacterium tuberculosis* infection induces type I IFN expression in human and mouse macrophages and myeloid dendritic cells (31–38). The serine/threonine kinase, TANK-binding kinase 1 (TBK1), is a central driver of type I IFN expression in *M. tuberculosis*-infected host cells (33). TBK1 facilitates phosphorylation of interferon regulatory factors (IRFs), and IRF3 and IRF5 have been reported to facilitate type I IFN expression in mycobacteria-infected host cells (39). IFN-β expression requires collaboration of IRFs with other transcription factors including NF-κB, ATF-2, and c-Jun (40), emphasizing the complexity of host signaling events that shape type I IFN expression. Pattern recognition receptors (PRRs) that have been linked to TBK1 phosphorylation and type I IFN expression in response to mycobacterial infection include the Nod-like receptor NOD2, stimulator of interferon genes (STING) either directly or *via* activation of cyclic GMP-AMP synthase (cGAS), as well as Toll-like receptor (TLR) 4 (Figure 1).

### The Mycobacterial Type VII Secretion System ESX-1 Facilitates Activation of Cytosolic Surveillance Pathways and Type I IFN Expression

Early studies established that induction of type I IFN responses by *M. tuberculosis* was greatly diminished if the genomic region of difference-1 (RD-1), the RD-1 encoded type VII ESAT-6 secretion system 1 (ESX-1), or its key components were deleted (33, 34, 39). The ESX-1 secretion system is a major virulence determinant of *M. tuberculosis* that mediates secretion of mycobacterial products that shape host responses to infection (41). ESX-1 may also directly affect phagosome membrane integrity (42). Co-localization studies with galectin-3, a marker of damaged membranes, and ubiquitin suggest that within 24 h of infection approximately 10% of *M. tuberculosis* bacteria are in contact with the cytoplasm and that this is dependent on ESX-1 (43). Compromised phagosomal membranes facilitate interactions of *M. tuberculosis* and its products with the cytosol fostering recognition by cytosolic surveillance pathways (33, 35, 39, 44, 45).

NOD2 has been implicated as a cytosolic sensor of *M. tuberculosis* that drives IFN-α and IFN-β expression upon infection (39, 46). Polyubiquitination of the NOD2-effector RIP2 was significantly diminished upon infection with ESX-1-deficient *M. tuberculosis* when compared to wild-type bacteria, identifying RIP2 as a major downstream effector of NOD2 in the recognition of *M. tuberculosis*. The unusual mycobacterial *N*-glycolylated muramyl dipeptide is a driver of NOD2-mediated type I IFN responses *via* RIP2 and IRF5, but not IRF3 (39). Reports addressing the role of NOD2 in the host control of *M. tuberculosis* infection in the mouse model *in vivo* have returned varying results. Whereas NOD2-deficient (*Card15*^−/−^) mice were not impaired in their ability to control infection with *M. tuberculosis* in two independent studies (47, 48), one study reported impaired T helper 1 (Th1) responses associated with slightly impaired survival and a modest increase in bacterial burden late during infection (48). Similarly, reports on human polymorphisms in the *NOD2* gene and susceptibility to, or protection from TB have returned varying results and will require further validation (49–53).

NOD2- and RIP2-deficiency only partially ablated *M. tuberculosis*-induced IFN-β and IFN-α expression (39), suggesting that additional molecular pathways of type I IFN induction exist. Several recent independent studies identified cGAS and STING as central drivers of IFN-β expression in human and murine macrophages during *M. tuberculosis* infection (35, 37, 38, 54). STING contains four transmembrane domains that anchor it into the endoplasmic reticulum membrane, as well as a large C-terminal domain involved in binding of TBK1 and IRF3. Binding of one molecule of cyclic di-nucleotide of either host or bacterial origin to STING dimers facilitates TBK1 and IRF3 activation (55). Endogenous 2′3′-cyclic GMP-AMP (cGAMP) is a second messenger generated by cGAS upon binding of cytoplasmic DNA. This cytoplasmic surveillance mechanism is a major driver of STING activation and type I IFN responses. Both cGAS- and STING-deficient human and mouse macrophages are impaired in their ability to express IFN-β in response to *M. tuberculosis* infection (35, 37, 38, 54). The underlying mechanisms that trigger this pathway are under intense investigation. It has been suggested that mycobacterial DNA gains access to the host cell cytoplasm and directly binds to cGAS, a process facilitated by ESX-1-dependent phagosome damage (37, 38). Others reported that the extent of macrophage IFN-β expression in response to different *M. tuberculosis* isolates correlated with mitochondrial stress, with no observable differences in the proportions of bacteria associated with damaged phagosomes (43). These observations suggest that the extent of mitochondrial DNA released during *M. tuberculosis* infection may contribute to cGAS-dependent activation of type I IFN expression by macrophages and determine the extent of the type I IFN response (43). Further amplification of STING-driven type I IFN expression may occur in uninfected bystander cells through shuttling of cGAMP through tight junction proteins (37, 56).

In addition, bacterial pathogens generate cyclic di-nucleotides that can activate STING independent of cGAS (55). The *M. tuberculosis* di-adenylate cyclase (disA or dacA) synthesizes c-di-AMP, which triggers IFN-β expression in macrophages and dendritic cells through STING activation, either through direct binding or *via* engagement of DEAD-box helicase 4 (DDX4) (35, 55, 57, 58). IFN-β expression in response to *M. tuberculosis disA* mutant bacteria was reduced compared to wild-type bacteria. Conversely, *disA* overexpression in *M. tuberculosis* CDC1551 and Erdman enhanced IFN-β expression (35). The discovery that *M. tuberculosis* expresses a phosphodiesterase, cdnP, that not only cleaves bacterial c-di-AMP and c-di-CMP but also the host-derived STING activator 2′3′-cGAMP (59, 60), is in keeping with the remarkable adaptation of this bacterium to its intracellular niche within the host. Accordingly, a *cdnP M. tuberculosis* transposon mutant induced elevated IFN-β, whereas *cdnP* overexpression impaired IFN-β expression by macrophages (60). Of note, the elevated IFN-β response elicited by *cdnP* deficient *M. tuberculosis* bacteria was independent of NOD2 (59), indicating that these cytoplasmic detection pathways are operating in parallel. Importantly, however, modulation of *disA* and *cdnP* expression did not only affect type I IFN expression but also pro-inflammatory cytokines like TNF, IL-6, and IL-1 (35, 60). These cytokines are important for the immune control of *M. tuberculosis* infection and can counter-regulate type I IFN expression (discussed below). This impact on the balance of key cytokines that are essential to control *M. tuberculosis* infection *in vivo* may account for the enhanced or attenuated virulence of the respective *M. tuberculosis* mutants *in vivo* (35, 59, 60). Moreover, the ability and extent of c-di-AMP production by *M. tuberculosis*, as well as cGAS and STING activation in *M. tuberculosis*-infected macrophages have been linked to autophagy (35, 38, 54). It will be valuable to further investigate molecular mechanisms of the engagement of pro-inflammatory cytokine responses and cell autonomous defense mechanisms by mycobacteria-derived c-di-AMP, which may involve other c-di-AMP-sensors such as DDX4 (35, 61) and RECON (62).

Despite the profound contributions of cGAS and STING to *M. tuberculosis*-induced type I IFN responses and autophagy activation reported by *in vitro* studies (35, 37, 38, 45, 54), bacterial burden in infected organs of mice deficient in cGAS or STING expression was comparable to wild-type controls (38, 54). One study reported impaired survival of cGAS- but not STING-deficient mice late during the chronic phase of infection (>100 days) (54). Whether this is linked to STING-independent functions of cGAS such as impaired autophagy (54, 63) remains to be established. Moreover, roles for DDX4 (61) and RECON (62) in regulating host responses and pathogen control during *M. tuberculosis* infection remain to be defined.

### ESX-1-Independent Induction of Type I IFNs in Mycobacterial Infection

While the data discussed above support a central role for the ESX-1 secretion system in the induction of type I IFN expression in *M. tuberculosis*-infected host cells, there is compelling evidence for mycobacteria-induced type I IFN responses in the absence of ESX-1. Type I IFN expression in response to ESX-1-deficient *M. tuberculosis* strains is reduced but not ablated compared to WT mycobacteria (38, 39). Moreover, the attenuated *Mycobacterium bovis* strain Bacillus Calmette–Guérin (BCG) lacks the RD-1 region including the genes that encode the ESX-1 secretion system (64). Yet, BCG induces type I IFN expression in both human and mouse primary macrophages, albeit to a significantly lesser degree than virulent *M. tuberculosis* (34, 35). IFN-β expression in BCG-infected macrophages was significantly reduced in *Sting^−/−^* cells, indicating STING-driven type I IFN expression. Moreover, overexpression of *disA* in BCG enhanced IFN-β expression compared to wild-type BCG (35). These data suggest that mycobacteria-derived dinucleotides may gain access to the cytosol in the absence of ESX-1 and induce IFN-β expression *via* activation of STING. It seems plausible that bacteria-derived dinucleotides may also activate STING and type I IFN expression in uninfected bystander cells by passing the cell membrane or through gap-junction *trans* signaling as proposed for cGAMP (37, 56). The extent to which mitochondrial stress responses may contribute to this STING-dependent type I IFN response (43) remains to be established.

Interactions of bacterial or viral components with specific TLRs can drive type I IFN expression. TLRs are type I transmembrane proteins that localize to the cell membrane or endosomal membranes. They sense microbial components in the extracellular environment or internalized materials in the “extracellular” space of intracellular trafficking compartments, but not in the cytoplasm (65). Dimerization of TLRs upon ligand binding facilitates recruitment of adaptor proteins and the initiation of intracellular signaling (66, 67). MyD88-dependent signaling from endosomal TLR7, TLR8, and TLR9 activates IRF-7 and results in the expression of type I IFNs (68). TLR4 internalization into endosomes upon stimulation with lipopolysaccharide engages TRIF and TRAM, facilitating IKKε and TBK-1 activation. Subsequent phosphorylation of IRF-3 leads to the expression of type I IFNs (69, 70). Recent data suggest that TLR2 may similarly induce TRIF- and TRAM-dependent signaling from endosomes (71). Initial studies concluded that TLRs are not a major driver of type I IFN responses during *M. tuberculosis* infection. *Ifnb1* expression by mouse macrophages infected with *M. tuberculosis* H37Rv was independent of TLR2, exhibited a minor defect in *Trif^−^*^/^*^−^* cells, and appeared to be negatively regulated by TLR4 (comparison of C3H/HeN versus C3H/HeJ) and MyD88 (33). A separate study showed that macrophage *Ifnb1* expression upon infection with the *M. tuberculosis* clinical isolate 1254 was equivalent between wild-type cells and macrophages deficient in *Tlr4, Tlr2, Tirap, Myd88*, or *Trif* (32). In contrast, TLR4-driven *Ifnb1* expression induced by the lineage 2 *M. tuberculosis* isolate BTB 02-171 was associated with TLR4-dependent enhanced virulence in mice (72, 73). While the TLR4 ligand in strain BTB 02-171 remains to be identified, these observations invite speculation that TLR4 signaling in this context may occur from maturing mycobacteria-containing phagosomes or endosomal compartments during infection. Whether such signaling engages TRIF, and/or also occurs downstream of other TLRs implicated in human susceptibility to *M. tuberculosis* infection, remains to be established. These findings encourage investigations into the TLR-activating properties of *M. tuberculosis* isolates dominant in populations or ethnic groups that show associations of TB susceptibility or severity with polymorphisms in TLR4 (74, 75), TLR2 and its co-receptors (76–81), as well as TLR8 (80, 82) and TLR9 (83). Comparisons with populations where such associations cannot be established (63, 84–87) will be of great value.

## Consequences of Type I IFN Signaling During *M. tuberculosis* Infection

The IFN gene signatures reported by the landmark study by Berry et al. (3) and subsequently several other studies firmly establish that IFNs flavor the peripheral immune response in patients with active TB. It is important to note that both type I and type II IFNs are implicated in driving these IFN signatures, especially those attributable to STAT1 homodimerization-stimulated gamma-IFN activation sequence (GAS)-regulated gene expression, which occurs in response to both type I and type II IFNs (88).

### Impact of Type I IFN Signaling in Mice and Humans on Pathogen Control and Disease Outcome

#### Mice

The functional consequences of type I IFN signaling in the context of *M. tuberculosis* infection are incompletely understood. The evidence so far suggests that the impact of type I IFN signaling on host resistance and disease severity are determined by the immune competence of the host, with contributions by the bacterial strain. The degree of *M. tuberculosis*-induced IFNα/β expression in mice has been correlated with the virulence of *M. tuberculosis* strains (43, 89). *Ifnar1*-deficiency in mice with a *M. tuberculosis*-susceptible genetic background (A129, 129S2) enhanced host survival upon infection with the high type I IFN-inducing hypervirulent HN878 strain but also the low IFN-inducing strain CDC1551 (90, 91). *Ifnar1*-deficiency also partially rescued accelerated mortality in the highly susceptible *Il1r*^−/−^ mice infected with the moderate type I IFN-inducer, H37Rv (92). These data suggest that endogenous type I IFN signaling impairs host resistance to *M. tuberculosis* infection in susceptible hosts. A study in C57BL/6 mice, a mouse strain more resistant to *M. tuberculosis* infection, reported equivalent survival rates between *Ifnar1*^−/−^ and C57BL/6 mice infected with the hypervirulent HN878 and other *M. tuberculosis* strains, despite diminished bacterial burden in the lungs of infected *Ifnar1*^−/−^ mice (93). Other studies compared C57BL/6 and *Ifnar1*^−/−^ mice during infection with H37Rv and the hypervirulent, high type I IFN-inducing BTB 02-171 strain for 70 and >200 days of infection. As neither C57BL/6 wild-type nor *Ifnar1*^−/−^ mice succumbed to the infection during these time frames, the impact of *Ifnar1* single deficiency on survival upon infection with these *M. tuberculosis* strains remained unclear (73, 94). Of note, *Ifnar1*^−/−^ mice on C57BL/6 background showed diminished bacterial burden and pathology as well as a minor survival advantage during long-term infection (>200 days) with *Mycobacterium africanum* (95). While *M. africanum* is a poor inducer of type I IFN responses (43), these data suggest that type I IFN signaling may subvert effective long-term host control of this infection in a relatively resistant host.

Administration of neutralizing anti-IFNα/β antibodies to resistant B6D2/F1 mice prior to and upon infection with *M. tuberculosis* HN878 provided a long-term survival benefit, associated with lower IFN-α expression and STAT1 activation in lung tissue, albeit without significant effects on bacterial burden (90). This suggests that pre-existing type I IFN responses in the host at the time of exposure may influence the outcome of *M. tuberculosis* infection. This is supported by the observation that pre-infection of mice with influenza A virus accelerated host death (>160 days of *M. tuberculosi*s infection). This was associated with exacerbated inflammation and some transient early and late elevation of bacterial burden, which was abrogated in *Ifnar1*^−/−^ mice (96). Moreover, artificial exacerbation of type I IFN responses in *M. tuberculosis*-infected resistant mice, e.g., by intranasal administration of IFNα/β or the stabilized synthetic TLR3 agonist pICLC, significantly impaired host survival, exacerbated lung inflammation, and impaired the host’s ability to restrict *M. tuberculosis* (89, 92, 97).

While the reasons for variable effects on bacterial burden and host survival in the abovementioned studies remain to be elucidated, the evidence gathered through genetic and exogenous modulation indicate detrimental effects of type I IFN signaling in mouse models of *M. tuberculosis* infection across a spectrum of host genetic backgrounds and mycobacterial strains (Table 1). However, there is evidence that type I IFN signaling exerts some protective effects in the absence of IFN-γ. Mice that lack the IFN-γ receptor are highly susceptible to *M. tuberculosis* infection, as reflected by poor host control of the bacteria, extensive pathology, and accelerated death. Additional *Ifnar1* deficiency in *Ifngr*^−/−^ mice further impaired survival suggesting that type I IFN signaling played a non-redundant protective role relatively early during infection with *M. tuberculosis* H37Rv and BTB 02-171 that was only apparent in the absence of IFN-γ signaling (73, 94). These observations suggest that the relative balance of type I and type II IFN functions defines host control of *M. tuberculosis* and pathology during the infection. It is worth noting that in mice infected with *M. bovis* BCG, *Mycobacterium avium*, and *Mycobacterium smegmatis*, type I IFNs appear to have protective roles (98–100), encouraging further investigations into the functional contributions of type I IFNs to host protection across a wider spectrum of mycobacteria.

**Table 1:** Perturbations of type I IFN signaling in mouse models and consequences for infection with bacteria of the *Mycobacterium tuberculosis* complex.

Mouse strain/Mtb susceptible (S) or resistant (R)	Type I IFN perturbation	Mycobacteria	Type I IFN response	Bacterial burden	Pathology	Survival	Reference
C57BL/6 (R)	*Ifnar1*^−/−^	H37Rv	Reduced IFN-β in lung homogenate 18 and 25 days p.i.	Comparable to WT on days 18 and 25 p.i.	n.d.	No mortality in WT and *Ifnar1*^−/−^ (day 70 p.i.)	(94)

	*Ifnar1*^−/−^	BTB 02-171	n.d.	Comparable to WT on days 24 and 27 p.i.	Comparable to WT at day 27 p.i.	No mortality in WT and *Ifnar1*^−/−^ (day 50 p.i.)	(73)

	*Ifnar1*^−/−^	Erdman	n.d.	Comparable to WT on days 1, 10, and 21 p.i. in the lungs. Reduced bacterial burden in spleen on days 10 and 21 p.i.	n.d.	n.d.	(33)

	*Ifnar1*^−/−^	*Mycobacterium africanum* GM041182	n.d.	Reduced bacterial burden in lungs on day 292 p.i. in *Ifnar1*^−/−^ mice	Pathology diminished in *Ifnar1*^−/−^ compared to WT mice	*Ifnar1*^−/−^ mice survived 292 days p.i., small number of WT mice succumbed >200 days p.i.	(95)

	Poly-ICLC (i.n.) twice weekly for 28 days, starting (a) 1 day or (b) 4 months post infection	H37Rv	(b) Poly-ICLC increased *Ifnb* and *Ifna* expression in lungs of WT mice; levels equivalent between poly-ICLC and poly-ICLC+Mtb	(a) and (b) increased bacterial burden in lungs. Was prevented in *Ifnar*^−/−^ mice	(a) and (b) increased lung pathology	(b) reduced survival	(97)

	Intranasal influenza A virus (a) 28 days before or (b) 1 and 14 days (2 different IVA subtypes) after Mtb infection	H37Rv	n.d.	Slightly elevated lung bacterial burden (a) 120 days (b) 27 days post Mtb infection	(a) increased lung inflammation on day 120 post Mtb infection	(a) impaired survival of mice >160 days	(96)
				(a) No effect on lung Mtb burden on days 28/31 and 63 post Mtb infection			

B6D2/F_1_ (R)	Purified mouse IFNα/β (5 days/weeks for 4 weeks) post infection	HN878	n.d.	Higher bacterial burden in lungs at day 28 for HN878 + IFNα/β compared to HN878 only mice	n.d.	IFNα/β administration significantly impaired survival	(89)

	Anti-IFNα/β (A1AB/5; i.p. every 48 h for 4 weeks starting 24 h prior to Mtb)	HN878	Lung IFN-α mRNA at d28 p.i. slightly elevated for anti-IFNα/β treated mice	Equal bacterial burden in lungs	n.d.	Enhanced survival of anti-IFNα/β treated mice	(90)

129 (S)	*Ifnar1*^−/−^	H37Rv	n.d.	Slightly decreased lung bacterial burden in *Ifnar1*^−/−^ mice 21 days p.i.	Diminished pathology in *Ifnar1*^−/−^ mice in *Ifnar1*^−/−^ mice 21 days p.i.	Most WT mice died within 40 days p.i., all *Ifnar1*^−/−^ mice survived (d70)	(91)

	*Ifnar1*^−/−^	HN878 CDC1551	n.d.	n.d.	n.d.	Small survival benefit for *Ifnar*^−/−^ infected with HN878; *Ifnar*^−/−^ survived significantly longer with CDC1551 infection compared to HN878	(90)

	*Ifnar1*^−/−^	H37Rv HN878 CSU123 CDC1551 Erdman-KO1	n.d.	Reduced bacterial burden in lungs of *Ifnar1*^−/−^ mice compared to WT mice (>25 days p.i.) for all Mtb strains	Increased lung pathology in WT mice infected with HN878 compared to H37Rv, CDC1551 or Erdman-KO1	Equivalent survival of *Ifnar1*^−/−^ (129 background) and C57BL/6 WT mice during infection with HN878, CDC1551, and Erdman-KO1	(93)

C57BL/6/129 (S)	*Ifnar1*^−/−^	Erdman	n.d.	Slightly increased lung bacterial burden at 10, 20, and 40 days p.i. compared to WT	n.d.	n.d.	(101)
				Comparable bacterial burden at 80 days p.i.			

*Il-1r*^−/−^ on C57BL/6 (S)	*Ifnar1*^−/−^	H37Rv	n.d.	n.d.	n.d.	*Il-1r*/*Ifnar1*^−/−^ partially rescued survival of highly susceptible *Il1r1*^−/−^ mice but still impaired compared to WT and *Ifnar1*^−/−^; no difference between WT and *Ifnar1*^−/−^ until day 80 p.i.	(92)

*Ifngr1*^−/−^ on C57BL/6 (S)	*Ifnαr1*^−/−^	H37Rv	Slightly elevated IFN-β protein in lung homogenate of *Ifngr/Ifnar1*^−/−^ compared to *Ifngr*^−/−^ mice 25 days p.i.	Comparable bacterial burden in lung and MLN in *Ifngr*/*Ifnar1*^−/−^ and *Ifngr*^−/−^; both higher than WT and *Ifnar1*^−/−^ 25 days p.i., no difference between WT and *Ifnar1*^−/−^	Severe pathology in *Ifngr*^−/−^ even more exacerbated in *Ifngr*/*Ifnar1*^−/−^, both higher than WT	*Ifngr*/*Ifnar1*^−/−^ succumbed approx. 7 days earlier than *Ifngr*^−/−^	(94)

	*Ifnar1*^−/−^	BTB 02-171	n.d.	Lung bacterial burden in *Ifngr*/*Ifnar1*^−/−^ mice increased compared to *Ifngr*^−/−^; both higher than WT and *Ifnar1*^−/−^ 24 and 27 days p.i., no difference between WT and *Ifnar1*^−/−^	Severe pathology in *Ifngr*^−/−^ even more exacerbated in *Ifngr*/*Ifnar1*^−/−,^ both higher than WT and *Ifnar1*^−/−^	*Ifngr*/*Ifnar1*^−/−^ succumbed approx. 3 days earlier than *Ifngr*^−/−^; no mortality in WT and *Ifnar1*^−/−^ (until day 50 p.i.)	(73)

*Ifnar1, interferon alpha receptor 1; Ifngr, interferon gamma receptor; Il-1r, interleukin 1 receptor; WT, wild-type; Mtb, M. tuberculosis; IFN, interferon; p.i., postinfection; i.n., intranasal; n.d., not determined; Poly-ICLC, polyinosinic-polycytidilic acid and poly-l-lysine double stranded RNA*.

#### Humans

The evidence obtained from mouse models of *M. tuberculosis* infection suggests that in the immune competent host, type I IFN signaling impedes the host’s ability to limit lung pathology and/or bacterial replication, to the benefit of the pathogen. However, there are several clinical case reports that inhaled or subcutaneous administration of IFN-α to tuberculosis patients, in conjunction with co-administration of antimycobacterial antibiotics, improved clinical symptoms (Table 2). It is important to note that these patients were selected due to failure of conventional treatment options and/or recurrent disease, and often received IFN-α late in their infection. Underlying unidentified immune deficiencies may thus have been a confounding factor. Moreover, reports of therapeutic effects of IFN-α administration to patients that presented with non-tuberculous mycobacterial infections suggest contributions of type I IFNs to the antimycobacterial defense in humans in the context of genetically manifested IFN-γ signaling deficiencies (102, 103). The clinical observations in IFN-γ signaling deficient patients are somewhat reminiscent of the studies in *Ifngr*^−/−^*Ifnar1*^−/−^ mice (73, 94). This encourages studies to enhance our understanding of the complex interplay between type I and type II IFN during mycobacterial infection and how this may be harnessed therapeutically in the context of compromised IFN-γ/IFNGR signaling. Recombinant IFN-α is commonly used in the treatment of chronic viral hepatitis. While some studies suggest that long-term administration of recombinant IFN-α does not bear a major risk for TB reactivation (104), case reports of TB reactivation associated with IFN-α therapy demand caution in the clinical management of co-infected individuals (105–109). Thus, detailed assessment of the direct and indirect roles of endogenous and therapeutically administered type I IFNs in the control of latent *M. tuberculosis* infection is an important area of future pursuit.

**Table 2:** Impact of type I IFNs in patients with mycobacterial infections.

Patients	TB	Comorbidity	Treatment	Outcome	Reference
	MDR-TB, XDR-TB				
	Non-tuberculous mycobacteria				
5 patients with advanced pulmonary cavitary disease, extensive drug resistance, chronic treatment failure	XDR-TB	–	Recombinant human IFN-α-2b subcutaneous injections weekly for 12 weeks in combination with anti-TB chemotherapy	2/5 clinical improvement, 1 year minimum six negative sputum cultures	(110)
				1/5 transient clinical improvement, smear negative but culture positive	
				2/5 not responsive	

7 patients non-responsive to second line chemotherapy after 6 months	MDR-TB	–	Aerosolized human IFN-α lymphoblastoid three times weekly for 9 weeks in combination with anti-TB chemotherapy	5/7 sputum smear negative but culture positive	(111)
				2/7 reduced sputum burden and remained culture positive	
				Bacterial burden increased in all after IFN-α treatment stopped	

20 patients, drug-sensitive pulmonary TB	TB	–	10 patients with anti-TB chemotherapy vs. 10 patients with anti-TB chemotherapy + aerosolized IFN-α three times weekly for 2 months	Fever, bacterial load in sputum smears and high-resolution computer tomography abnormalities showed earlier resolution in IFN-α treated group	(112)

11 patients, pulmonary TB, isoniazid, and rifampin resistance	MDR-TB	–	6 patients with anti-TB chemotherapy vs. 6 patients with anti-TB chemotherapy + s.c. IFN-α three times weekly for 8 weeks	8 weeks: 5/5 sputum smear negative in IFN-α group	(113)
				6 months: 2/5 sputum negative in IFN-α group	
				0/6 sputum negative in control group at both time points	

48 years old male; previous treatment failure	TB	Diabetes mellitus	Co-administration of IFN-α-2a i.m. weekly for 8 months with anti-TB chemotherapy	Afebrile and body weight gain after 2 weeks, and radiological improvement after 2 months	(114)
				Radiological status stable and sputum cultures negative after 4 years	

4 patients with disseminated disease; patients A–C had early childhood mycobacterial disease onset	Patient A: *Mycobacterium avium* complex (MAC), *Mycobacterium kansasii, Mycobacterium fortuitum*	Complete or partial IFNGR1 signaling deficiencies	Patient A: IFN-α2b three times weekly subcutaneously for 3 months	Patient A: reduction of pulmonary disease, lesions resolved after 1 year	(103)
Patient A: 22 years old female	Patient B: MAC, *Mycobacterium abscessus*	Patient B: B-cell lymphoma	Patient B: IFN-α2b 3 times weekly subcutaneously, then pegylated IFN-α	Patient B: deceased at 20 years of age, but biopsies showed no mycobacteria	
Patient B: 19 years old male	Patient C: *Mycobacterium bovis* BCG, MDR *M. bovis*		Patient C and D: alternating doses of IFN-γ and IFN-α2b subcutaneously three times weekly; all in conjunction with individualized antibacterial treatment	Patient C: gained weight, lesion healed, pleural mass decreased	
Patient C: 7 years old male with Bacillus Calmette–Guérin (BCG) immunization at birth	Patient D: *M. fortuitum, MAC*			Patient D: no new infections	
Patient D: 52 years old female					

12 months old female disseminated disease	MAC	Complete IFNGR1 deficiency and complete loss of functional response to exogenous IFN-γ	Co-administration of IFN-α-2b subcutaneous injections three times weekly and antimycobacterials	Mycobacteremic but clinically stable with continuation of multi-drug regimen at 58 months of age	(102)

*TB, tuberculosis; MDR, multi-drug resistant; XDR, extensively drug resistant; IFN, interferon; IFNGR1, interferon-gamma receptor 1; s.c., subcutaneous; i.m., intramuscular*.

It is obvious that our understanding of the contributions of type I IFN signaling to host control of mycobacterial infections is incomplete. Parallels between mouse models and human patients are emerging, but remain limited. In-depth knowledge of type I IFN responses and their contributions to host control in other model systems may be required to bridge important gaps between human and mouse immune pathogenesis during *M. tuberculosis* infection. The non-human primate model where IFN signaling transcriptional signatures are also highly associated with active TB disease may offer valuable opportunities (115). Confounding factors that require careful consideration in future studies include the impact of host genetic susceptibility, the host’s type I IFN status at the time of infection, the temporal contributions of type I IFNs in active and latent infection, the site of infection, and the infecting mycobacterial species. Detailed understanding of the timing and nature of the interactions between type I and type II IFNs may help to more clearly define intervention strategies and risk factors in the management of mycobacterial infections in patients with (known or unknown) underlying immune deficiencies and co-infections.

### Mechanistic Insights into How Type I IFNs Regulate Immune Responses during *M. tuberculosis* Infection

**Figure 2:**
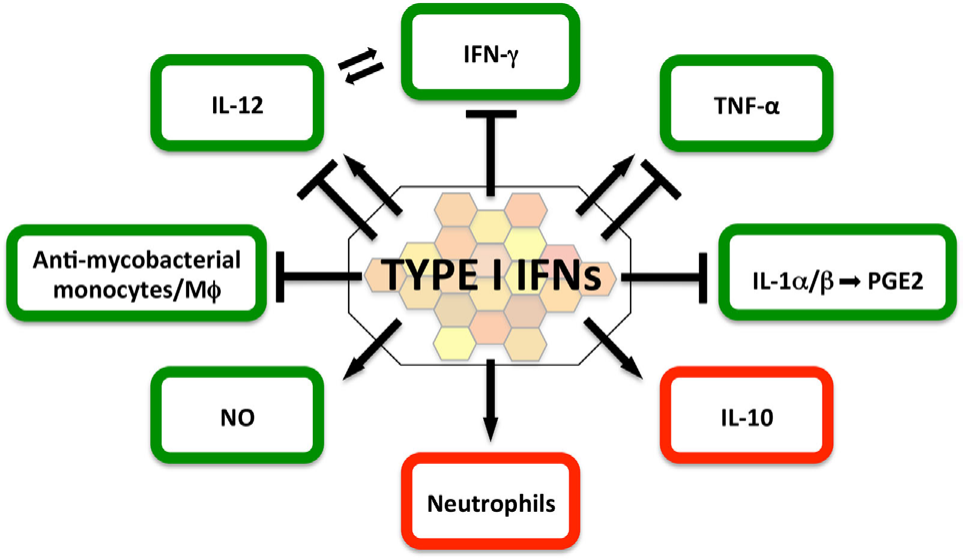
Impact of type I IFNs on protective (green) and detrimental (red) host responses in the immune-competent *Mycobacterium tuberculosis*-infected host. The current literature suggests that in the immune-competent host, type I IFNs suppress host-protective IFN-γ and IL-1α/β responses, as well as the recruitment of mycobacteria-restricting monocytes/macrophages. In contrast, type I IFN signaling can directly induce NO production, a contributor to effective antimycobacterial host defense. The evidence for the contributions of type I IFNs to host-protective interleukin-12 and TNF responses is currently ambiguous and may be determined by timing, dose, and source of the type I IFN response. Type I IFNs are a driver of IL-10, a cytokine that impairs antimycobacterial immune responses during all stages of infection. Type I IFN signatures in patients have been associated with neutrophils and studies in mouse models of heightened *M. tuberculosis* susceptibility suggest that type I IFN signaling facilitates infection of permissive neutrophils, which have been associated with damaging tissue pathology.

In light of the potential implications for host-directed therapies in TB, the molecular and cellular mechanisms by which type I IFNs suppress, or in some contexts promote, effective immune control of *M. tuberculosis* are the subject of intense investigation and discussion (2, 116, 117) (Figure 2).

#### Myeloid Antimicrobial Defense

Independent studies in different mouse models suggest that type I IFN signaling during *M. tuberculosis* infection defines the nature of the early myeloid cell infiltrate into the lung, which may favor bacterial replication due to a diminished ability to restrict intracellular mycobacteria (91, 97, 118). Type I IFNs impair the IFN-γ-induced human and mouse macrophage control of *M. tuberculosis* and other mycobacteria (119–121). At least in human macrophages, this impaired ability to control intracellular mycobacteria is likely facilitated through the IL-10-mediated inhibition of the IFN-γ-driven vitamin D3/cathelicidin/defensin beta 4A antimicrobial defense pathway (120). The interferon-stimulated gene (ISG) ISG-15 has recently been implicated in promotion of early intracellular *M. tuberculosis* replication in mice, but seems to have protective roles later during infection (122). This later role supports findings in ISG15-deficient patients with mycobacterial disease that suggest that secreted ISG15 promotes IFN-γ secretion (123). Of note, ISG15 expression was detectable in several human leukocyte subpopulations including T cells, NK cells, myeloid and plasmacytoid dendritic cells, monocytes, with the highest levels detected in granulocytes. Exogenous IFN-a enhanced *ISG15* expression most dramatically in granulocytes (123), which may provide a link for the functional importance of neutrophil-associated IFN-inducible gene signatures in humans with active disease (3). In contrast to the impaired IFN-γ-mediated macrophage control of mycobacteria, in the absence of IFN-γ signaling type I IFNs may facilitate the recruitment, differentiation and/or survival of myeloid cells that control *M. tuberculosis* to some extent. This may be due to type I IFN-mediated prevention of skewing of macrophages toward an alternatively activated phenotype (73, 94).

#### IL-12/IFN-γ and Th1 Response

High type I IFN responses in mice infected with hypervirulent HN878 are associated with diminished Th1 responses including lower IFN-γ production and proliferation by antigen-specific T cells (89, 90, 93). There is *in vitro* and *in vivo* evidence that type I IFNs exert these suppressive effects through impairment of myeloid-derived IL-12 production. In myeloid cells derived from *M. tuberculosis*-infected bone marrow chimeric mice, IFNAR-deficiency enhanced myeloid cell-derived IL-12p40 expression in response to re-stimulation with *M. tuberculosis* compared to wild-type cells (116). In murine *M. tuberculosis*-infected macrophages, exogenous addition of high concentrations of IFN-β suppressed IL-12 expression as well as IFN-γ-induced macrophage activation (121). Exogenously added type I IFNs also suppressed IFN-γ-induced cytokine responses in human monocytes (120, 124). Molecular mechanisms underlying type I IFN-mediated suppression of IL-12 responses include the induction of IL-10, downregulation of the IFN-γ receptor, and induction of protein arginine methyl transferase 1, a negative regulator of IFN signaling (120, 121, 124). However, diminished IL-12p40 release by *M. tuberculosis*-infected IFNAR^−/−^ macrophages has been reported (121), suggesting that in isolated macrophage cultures, endogenous type I IFN signaling facilitates IL-12p40 responses. Future studies may need to address similarities and potential differences in the effects of endogenously produced versus exogenously added type I IFNs both in cell culture as well as *in vivo*.

Exogenous addition of recombinant IFN-α to *in vitro* cultured T cell clones from TB patients enhanced the number of clones that produced IFN-γ (125) in line with observations that IFN-α can promote IFN-γ production by human CD4^+^ T cells (126). Thus, the overall impact of type I IFN signaling during mycobacterial infection may be a composite of differential effects on myeloid and lymphocyte cell functions. Mouse models that allow assessment of the impact of type I IFN signaling in specific cell subsets such as T cells (127) might prove valuable in dissecting the direct impact of type I IFNs on T cell functions during *M. tuberculosis* infection.

#### IL-10

Early studies associated higher virulence of *M. tuberculosis* isolates (defined by elevated bacterial burden and impaired survival of infected mice) with the capacity to elicit high type I IFN responses *in vivo*. This phenotype was accompanied by relatively lower pro-inflammatory cytokine responses (89, 90, 93). More recent data indicate that elevated virulence in the mouse model can be associated with induction of both high IFN-β and high pro-inflammatory cytokine responses (72). Association of elevated virulence with high type I IFN paired with high IL-10 levels may provide a more direct link between high type I IFN responses and impaired host control of the infection. Type I IFN signaling drives IL-10 expression by both *M. tuberculosis*-infected macrophages and CD4^+^ T cells in infected mice (121, 128). While there is great variability between observations made in different mouse strains and between different labs, several studies in mice suggest that IL-10 significantly impairs host control of *M. tuberculosis* infection during active, chronic, and latent infection (2). Overexpression of IL-10 in macrophages driven by the CD86 promoter led to increased bacterial burden and impaired survival of *M. tuberculosis-*infected mice without noticeable effects on T cell functions (129). In contrast, a recent study that utilized cell-specific deletion of *Il10* expression *in vivo* suggests that a reduction of bacterial burden in lungs of IL-10-deficient mice during the chronic phase of infection (day 60) is attributable to IL-10 produced by CD4^+^ T cells rather than myeloid or B cells. However, while lung CD4^+^ T cells from *M. tuberculosis*-infected *Ifnar1^−^*^/^*^−^* showed diminished *Il10* expression, lung bacterial burden in *Ifnar1^−^*^/^*^−^* mice was not decreased and did hence not mirror the reduction of lung bacterial load in *Il10^−^*^/^*^−^* and *Il10*^fl/fl^ CD4^Cre+^ mice (128). Instead, IL-27 receptor signaling was implicated to drive IL-10 expression by CD4^+^ T cells, in parallel to type I IFNs, and *IL27ra^−^*^/^*^−^* mice exhibited diminished lung bacterial burden similar to *Il10^−^*^/^*^−^* and *Il10*^fl/fl^ CD4^Cre+^ mice (128). Thus, cell-specific targeting of the cellular source of IL-10 that impairs host control of *M. tuberculosis* replication may be of interest for host-directed intervention but requires detailed characterization of the functionalities and impact of IL-10-producing host cell populations.

#### TNF-α

The increased risk of TB reactivation in individuals undergoing TNF neutralizing therapies for chronic inflammatory diseases underpins the central role for this cytokine in the host control of *M. tuberculosis* infection, which is further supported by mouse studies (2, 130). Macrophages deficient for IFNAR signaling showed diminished TNF expression in response to *M. tuberculosis* infection, which was interpreted as a promoting role for endogenous type I IFN responses in the *M. tuberculosis*-induced TNF response (121). However, an independent study reported no differences between IFNAR-deficient and WT macrophages (131). In contrast, exogenously added IFN-β suppressed TNF release by *M. tuberculosis*-infected macrophages (121). The impact of type I IFN signaling on the TNF response *in vivo* remains to be clearly defined. WT and IFNAR-deficient mice infected with the high type I IFN-inducing *M. tuberculosis* strain BTB 02-171 displayed equivalent lung *Tnf* mRNA expression (day 20 p.i.) (73). In contrast, decreased TNF concentrations were observed in lung homogenates of *M. tuberculosis*-infected IFNAR-deficient 129 mice (day 21 p.i.) (91) and a small decrease in lung TNF concentrations was also reported in *M. africanum*-infected IFNAR-deficient C57BL/6 mice (day 292 p.i.) (95). The reasons for the apparent differences between individual studies are not immediately obvious, but may be attributable to the different time points analyzed postinfection and the different models systems studied. These observations may indicate, however, that the concentration, source, and timing of the type I IFN response direct TNF responses in the context of *M. tuberculosis* infection.

#### IL-1, Eicosanoids, Nitric Oxide (NO), and Neutrophils

IL-1 signaling is an important driver of host resistance against *M. tuberculosis* infection in humans and mice (132–135). The underlying molecular mechanisms are emerging and TNF-driven antimicrobial activity, autophagy, as well as eicosanoid signaling have been implicated (92, 134, 136). Type I IFN signaling suppressed IL-1α and β production by macrophages *in vitro* and *in vivo* mouse infection (34, 121, 137). The type I IFN-mediated regulation of IL-1 expression by human and mouse macrophages was shown to be dependent on NO synthase 2 and IL-10, and was associated with enhanced IL-1Ra expression (121, 137).

Thus, type I IFN signaling regulates IL-1 responses through inhibition of ligand expression as well as receptor signaling. A third potential mechanism may be inferred from the observation that NO interference with NLRP3 inflammasome assembly impairs IL-1β processing by *M. tuberculosis*-infected macrophages (138). As endogenous and exogenously added type I IFNs drive NO production by *M. tuberculosis*-infected macrophages (32, 73), type I IFN signaling may also interfere with IL-1 processing. It is interesting to note that NO-mediated thiol nitrosylation inhibited assembly and activation of the NLRP3 but not AIM2 inflammasome (138). AIM2 has been reported to be dispensable for IL-1β production by murine dendritic cells infected with virulent *M. tuberculosis in vitro*. In contrast, AIM2 was required for IL-1β release induced by *Mycobacterium smegmantis, Mycobacterium fortuitum, Mycobacterium kansasii* and, to a lesser extent, the attenuated *M. tuberculosis* H37Ra (139). Intriguingly, infection of DCs with virulent *M. tuberculosis* prior to infection with *M. smegmatis* reduced the IL-1β release, which was associated with an impaired IFN-β response. The authors concluded that virulent *M. tuberculosis* actively suppressed AIM2 activation by a mechanism that required *M. tuberculosis* ESX-1 (139). Yet, AIM2-deficient mice infected intratracheally with a high dose of *M. tuberculosis* H37Rv displayed elevated bacterial loads in the lung and liver 4 weeks postinfection accompanied by exacerbated pathology, impaired IL-18 and IL-10 production, as well as an impaired antigen-specific CD4^+^ T cell-derived IFN-g response. The *Aim2*^−/−^ mice succumbed to infection between 5 and 7 weeks postinoculation (140). It is important to note, however, that deficiency in caspase 1 and 11, inflammasome effector caspases that cleave pro-IL-1β, does not impair host control of *M. tuberculosis* infection *in vivo* (133, 141). This suggests that alternative mechanisms of pro-IL-1 processing during *M. tuberculosis* infection *in vivo* exist, and that AIM2 contributions to the host control of *M. tuberculosis* infection may exceed processing of pro-IL-1β.

An emerging pathway of type I IFN-mediated suppression of effective immune control of *M. tuberculosis* occurs *via* dysregulation of IL-1-controlled eicosanoid lipid mediators (116). In mice, IL-1 receptor signaling enhanced the ratio of host-protective prostaglandin E2 over host-detrimental 5-lipoxygenase products such as lipoxin A4 (116). Type I IFN signaling counter-regulates this IL-1-driven process, and conversely, IL-1 signaling impairs the type I IFN response (92). Poor control of *M. tuberculosis* infection in various mouse models is associated with an extensive neutrophil response in the lung. Pathogen control and host survival can be improved by neutrophil depletion and *Ifnar1*-deficiency in a susceptible mouse strain, which is associated with a shift of intracellular *M. tuberculosis* burden from permissive neutrophils toward monocytes (91, 142, 143). Recent findings suggest that in a permissive environment, 12/15-lipoxygenase products drive neutrophil infiltration into infected lungs, which is associated with poor host restriction of mycobacterial replication in a nutrient-rich environment (143). In the absence of NO production (*Nos2^−/−^* mice), this process was driven by inflammasome-dependent IL-1 production (143) linking back to the inhibitory effects of NO on inflammasome assembly (138).

Associations of IL-1, type I IFN, eicosanoids, neutrophil infiltration, and SNPs in associated genes with disease severity and treatment responsiveness in human TB patients (92, 135, 143) underpin a dynamic cytokine network that balances the protective or detrimental roles of eicosanoid lipid mediators and neutrophil functions in *M. tuberculosis* infection. Pharmacologic targeting of eicosanoids has been proposed as a possible avenue for host-directed therapeutic intervention in TB (92).

## Concluding Remarks

As we continue to gain mechanistic insight into the interactions between *M. tuberculosis* and innate immune sensors, it is evident that multiple independent extracellular and intracellular surveillance pathways converge to drive type I IFN expression in infected host cells. One of the main questions remaining is which cells are the key producers of type I IFNs during *M. tuberculosis* infection *in vivo*. *In vitro* evidence strongly suggests that host cells that harbor *M. tuberculosis* bacteria such as macrophages and myeloid DCs are sources of type I IFN during infection. In the context of the close interactions between infected and uninfected cells within granulomas, the concept of dinucleotide-mediated STING-activation in bystander cells warrants further exploration as it may allow for type I IFN expression not only in myeloid cells but also in T and B cells. Moreover, mice infected with hypervirulent, high type I IFN-inducing HN878 harbored elevated numbers of plasmacytoid DCs (pDCs) in their lungs (93). Human peripheral blood pDCs responded with high IFN-α production to stimulation with high concentrations of mycobacterial DNA (144). However, *in vitro* cultured pDCs expressed little to no type I IFNs when co-cultured with *M. tuberculosis-* or *M. bovis*-BCG-infected myeloid DCs (144, 145). Advanced single cell functional profiling in TB granulomas and the employment of sensitive IFN reporter animal models are just two approaches that may provide a better understanding of the dynamics and cellular sources of type I IFN expression during *M. tuberculosis* infection both at the site of infection and in the periphery. Individual type I IFNs exhibit distinct functions in various homeostatic and pathological contexts, which has at least in part been attributed to varying ligand affinity, stability of the receptor complex, and unique signaling capacity of IFN-β through IFNAR1 (146–148). Thus, insights into the spatial and temporal expression of individual members of the type I IFN family may hold the key to providing context for some of the apparent opposing roles of type I IFN signaling deduced from model systems and patient observations. As the discoveries around blood gene expression signatures in patients with active TB disease are being explored for suitable biomarkers, they also raise questions related to TB pathophysiology. For example: are IFN signatures related to neutrophils in the periphery (3, 149) a natural consequence of *M. tuberculosis* infection in humans, or an indication of disease susceptibility of the individual due to a propensity to mount a neutrophil response upon encounter with *M. tuberculosis*? How reflective is the peripheral gene expression profile of the response mounted locally at the site of infection or within the relevant secondary lymphoid organs? Longitudinal analyses and immune profiling in at-risk individuals (8) will provide valuable insights, and suitable animal models are key for functional context. While type I IFN signaling may contribute to some aspects of the host defense against *M. tuberculosis* infection, it is clear that extensive type I IFN responses, e.g. in the context of viral infections (150), promote pathology to the detriment of the host. Nevertheless, the reported therapeutic benefits of type I IFN administration in conjunction with antimycobacterial antibiotics warrants detailed exploration to establish whether this is reflective of a patient immune status permissive to potential antimycobacterial activities of type I IFN or whether IFNs facilitate mycobacterial replication rendering them more susceptible to antibiotics. Understanding whether and when type I IFNs are “friend or foe” in the context of mycobacterial infections will define their place in the quest for tailored host-directed therapeutic interventions in TB.

## Author Contributions

All authors contributed to the critical review of the literature and writing of the manuscript. MD and AB developed the figures and tables and edited the manuscript.

## Conflict of Interest Statement

The authors declare that the research was conducted in the absence of any commercial or financial relationships that could be construed as a potential conflict of interest.
